# External stenting of vein grafts in coronary artery bypass grating: interim results from a two centers prospective study

**DOI:** 10.1186/s13019-021-01406-0

**Published:** 2021-04-12

**Authors:** Luca Paolo Weltert, Katia Audisio, Alessandro Bellisaro, Gianluca Bardi, Roberto Flocco, Ruggero De Paulis, Paolo Centofanti

**Affiliations:** 1 Heart Surgery Unit, European Hospital, 700, Via portuense, 00149 Rome, Italy; 2 Department of Statistics, Saint Camillus International University of Health and Medical Sciences, 8, Via di Sant’Alessandro, 00131 Rome, Italy; 3 Heart Surgery Unit, Mauriziano Hospital, 62, Largo Filippo Turati, 10128 Turin, Italy

**Keywords:** Coronary artery bypass grafting, Myocardial revascularization, Vein grafts, Vein graft disease, Venous external stent

## Abstract

**Background:**

previous studies evaluating external stents for saphenous vein grafts (SVG) in CABG were limited to on-pump isolated CABG and single grafting technique with one external stent per patient. The objective of this prospective study was to evaluate the safety and the short-term performance of external stents in a heterogeneous group of patients who underwent on- and off-pump CABG, single and sequential grafting.

**Methods:**

102 patients undergoing CABG were enrolled in two centers. All patients received internal mammary artery to the left anterior descending artery and additional arterial and/or venous grafts. In each patient, at least one SVG was supported with an external stent. Grafts’ patency and SVG lumen uniformity were assessed using CT angiography at a pre-defined time window of 6–12 months post procedure. All patients were prospectively followed-up via phone call and/or visit every 6 months for Major Adverse Cardiac and Cerebrovascular Events.

**Results:**

51 patients (50%) underwent off-pump CABG and 23 patients (23%) were grafted with bilateral internal mammary arteries. Each patient received one or more SVG grafted in a sequential technique (44%) or as a single graft (56%). All SVG were externally stented in 84% of patients and in 16% (*n* = 16) one SVG was stented and one remained unsupported. At 6–12 months, patency rates of LIMA, RIMA, externally stented SVG and none-stented SVG were 100, 100, 98 and 87.5% respectively. 90% of the externally stented SVG had uniform lumen compared to 37% of the non-stented SVG. Clinical follow-up was completed for all patients with a mean duration of 20 months (range 6–54 months). During follow up period, one patient experienced myocardial infarction due to occlusion of the LIMA-LAD graft and one patient experienced a transient ischemic attack.

**Conclusions:**

External stenting of SVG is feasible and safe in CABG setting which includes off pump CABG and sequential SVG grafting and associated with acceptable early patency rates.

**Trial registration:**

Study was registered at ClinicalTrials.gov. NCT01860274 (initial release 20.05.2013).

## Introduction

Coronary artery bypass grafting (CABG) remains the gold standard treatment for multivessel coronary artery disease [1]. Despite extensive clinical research, the long-term outcome of CABG is still limited by the poor longevity of saphenous vein grafts (SVG), the most commonly used type of conduit [2]. Although the root causes of SVG disease were discovered decades ago, these findings were not translated into the clinical setting in which only 50% of vein grafts are patent 10 years after CABG [3].

The accelerated SVG disease is attributed to early structural remodeling of the vein due to exposure to the hemodynamics of the arterial circulation and the development of intimal hyperplasia [4, 5]. Except for statins and beta- blockers, pharmacological attempts to mitigate vein graft disease have shown limited success [6]. Surgical approaches to reduce SVG disease, which were focused on optimization of the harvesting method, have shown that a no-touch technique leads to a significant increase in SVG patency compared to conventional harvesting [7].

Experimental studies demonstrated a significant effect of external stenting on the progression of vein graft disease post implantation [8, 9]. These findings were translated recently into the clinical setting and randomized trials have shown promising evidence regarding the effect of external stents on intimal hyperplasia and structural SVG remodeling (Fitzgibbon classification) up to 4.5 years after CABG [10–13].

However, previous reports regarding the benefits of external stenting were limited to a highly selective group of patients who underwent isolated on-pump CABG and single SVG grafting. The objective of our study was to evaluate the safety, technical feasibility and early outcome of external stenting in routine practice which includes heterogeneous group of patients undergoing on and off-pump CABG, single and sequential SVG grafting.

## Methods

### Patients

Patients undergoing elective surgical revascularization at two centers with at least one SVG were included in the study. Upon device availability in the hospital, consecutive patients were recruited by two senior surgeons (one in each hospital) who were trained with the implantation technique. The study was approved by the local ethics committee and all patients gave informed consent (NCT01860274). All patients underwent CABG with the internal mammary artery (IMA) to the left anterior descending artery (LAD) and additional arterial or venous grafts. In each patient, at least one SVG was supported with an external stent (VEST, Vascular Graft Solutions, Israel) according to surgeon’s discretion.

### Intraoperative

**Fig. 1:**
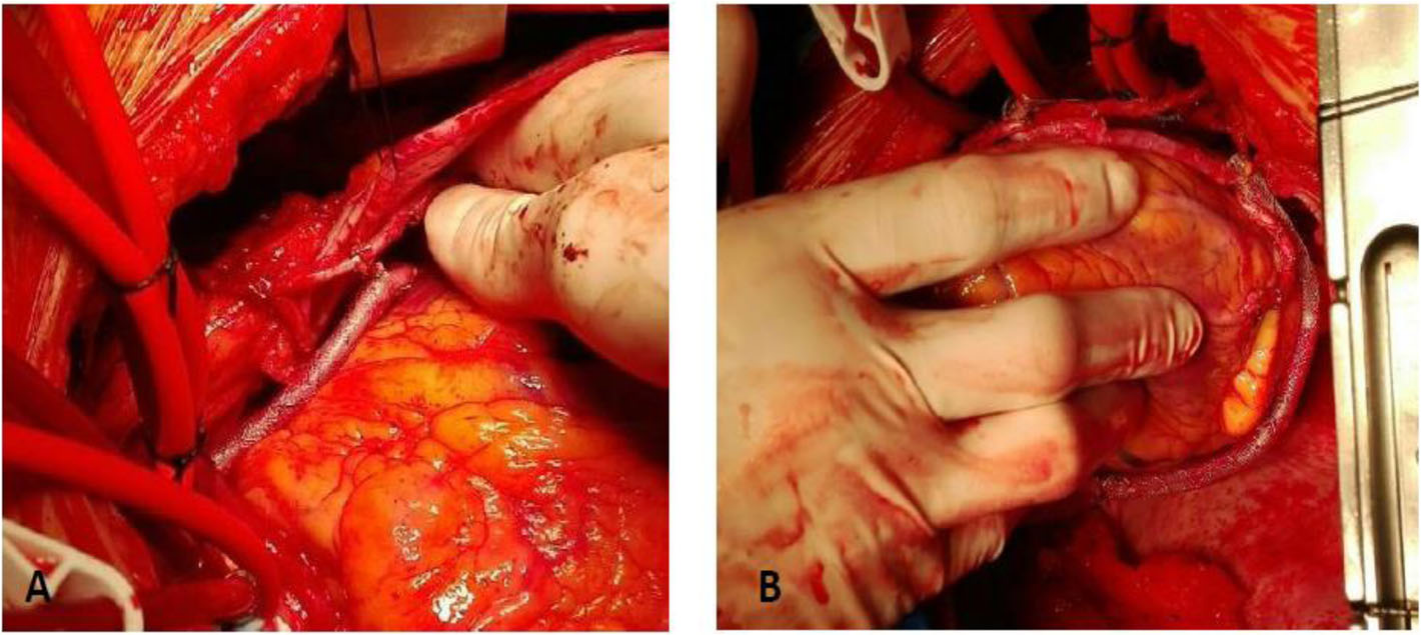
– VEST implantation (**a**) sequential SVG bypassed to the posterior descending artery (**b**) aortocoronary SVG segment bypassed to the ramus intermediate artery

In all cases, SVG harvesting was performed in a conventional open manner and all side branches were ligated with sutures or ties. Heparin was not given prior to SVG harvesting and the vein was stored in normal saline. During preparation, the vein was marked each 5 cm and its diameter was assessed using a dedicated measurement tool. After completion of the distal anastomosis, SVG length was accurately measured and an external stent model was chosen based on SVG length and diameter. The device was threaded over the vein and was manually expanded to cover the entire SVG leaving a 2-10 mm unsupported segment near the anastomoses. In case of multiple distal anastomoses, SVG length and diameter of each segment were assessed after completion of the distal anastomosis and the appropriate stent model was chosen to support the segment between the two distal anastomoses (Fig. 1). During off-pump CABG, if the SVG was dilated due to backflow after completion of the distal anastomosis, a vascular clamp was applied on the distal end of the SVG to allow deflation and enable stent expansion on a non-pressurized graft. The stent has radial elasticity and axial plasticity that enabled the surgeon to compress the stent in order to enable adequate evaluation/revision of the graft or the anastomosis with subsequent re-expansion. Prior to closing the chest, Transit Time Flow Measurement (TTFM) was applied to all grafts and corrective measures were taken as needed. In order to perform TTFM measurements, the proximal end of the stent was slightly compressed and then re-positioned after completion of TTFM measurement.

Assessment and optimization of postoperative coagulation state were performed using Point of Care Thromboelastography. After CABG, all patients were prescribed with statins, anti-platelets and beta blockers. In-hospital adverse events were recorded including death, myocardial infarction, stroke, transient ischemic attack and need for revascularization.

### CT angiography

**Fig. 2:**
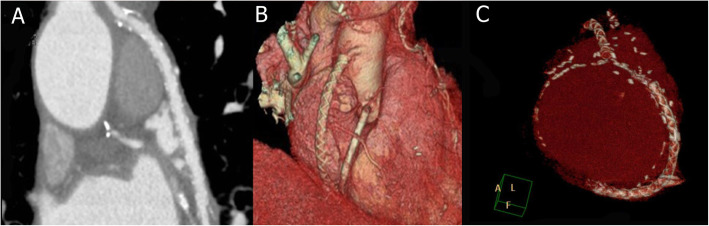
– CT angiography of externally stented SVG to the right coronary artery in a 2D (**a**) and 3D reconstruction (**b**), and a 3D reconstruction of externally stented sequential SVG to the ramus intermediate and the posterior descending arteries (**c**).

**Fig. 3:**
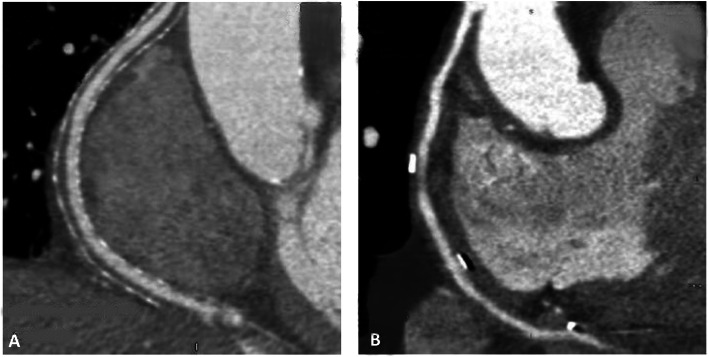
**–** Externally stented SVG to the right coronary territory demonstrating a uniform lumen (**a**) and unsupported SVG to the right coronary territory with a non-uniform lumen (**b**)

CT angiography (OPTIMA 660, GE, 64 slices) was performed to assess grafts patency and lumen uniformity 6–12 after CABG (Fig. 2). Oral nitroglycerin was given prior to the scan in order to achieve coronary vasodilatation; β-blocker (Esmololo, 1 fl) was also administered to patients with heart rate above 65 bpm. After defining the region of interest, 95 ml of an iodine based contrast agent (Ultravist 370) was injected at a flow rate of 5 ml/sec followed by a saline chaser bolus of 40 mL at 5 ml/sec, via a 20 gauge needle in the antecubital fossa. The gantry rotation time was 0.35 s, peak tube voltage was 120kVp, and current (mA) was adjusted per patient’s body weight. SVG were graded by an independent observer (radiologist) to be (1) patent with < 50% stenosis (2) patent with > 50% stenosis (3) occluded. In addition, as shown in Fig. 3, SVG were classified to have uniform lumen or lumen irregularities defined as lumen diameter variation > 0.5 mm.

### Clinical follow up

All patients will be prospectively followed-up via phone call and/or visit every 6 months for a period of 5 years. Major Adverse Cardiac and Cerebrovascular Events (MACCE), defined as the composite occurrence of all-cause mortality, myocardial infarction, revascularization, and stroke are recorded per each phone call or visit.

### Statistical analysis

Continuous data are expressed as mean and standard deviations or median and range after assessment of normality distribution. Categorical data are presented as absolute values and percentages. Assessment of normality of distribution was performed by Kolmogorov-Smirnov test.

## Results

Between September 2015 and December 2019, 102 patients that underwent CABG were enrolled in  this study. None of the patients had prior cardiac surgery and 26% of patients had prior percutaneous revascularization. Baseline demographics are presented in Table 1. All patients were maintained on aspirin during CABG and 96% of patients were receiving statins (20 mg Atorvastatin) at the time of operation.

**Table 1:** Baseline demographics

	Mean ± SD or n (%)
Age (yrs.)	68.5 ± 11
Gender (male)	89 (87%)
Weight (kg)	74 ± 12
Height (cm)	168 ± 7
Body Mass Index (kg/cm2)	26 ± 4
Ex smokers or current smokers	84 (82%)
Insulin dependent diabetes	28 (27%)
Hypertension	86 (84%)
Dyslipidemia	84 (82%)
Diffuse peripheral artery disease	13 (13%)
Vasculitis	0 (0%)
Prior cardiac surgery	0 (0%)
Prior percutaneous revascularization	27 (26%)
Prior stroke in the past 2 years	6 (6%)
COPD of at least moderate grade	23 (23%)
NYHA Class	2.2 ± 1.5
CCS Class	3 ± 2.2
Ejection Fraction	55 ± 7%
Creatinin (mg/dl)	1.07 ± 0.7
Euroscore	3.6% ± 3.8%,

*CCS* Canadian Cardiovascular Society, *COPD* chronic obstructive pulmonary disease, *NYHA* New York Heart Association, *SD* standard deviation

The revascularization strategy was determined by the surgeon and no change in the surgical plan was made intraoperatively due to the use of the external stent. 51 patients (50%) underwent off-pump CABG. All patients underwent CABG with IMA-LAD grafts and 23 patients (23%) received bilateral internal mammary artery (BIMA) grafting. In addition, each patient received one or more SVG grafted in a sequential technique with two or three segments (*n* = 45) or as a single conduit with one distal anastomosis (*n* = 57). In 84% of the patients (*n* = 86) all SVG were supported with an external stent and in 16% (*n* = 16) at least one SVG was stented and one remained unsupported. Grafts distribution according to the coronary target is presented in Table 2. External stent deployment was successful in all patients with no technical failure. Expansion of the stent to cover the entire SVG length did not damage the anastomosis or caused new bleeding from the suture sites. In patients undergoing on-pump CABG warm intermittent blood cardioplegia was used with an average cross-clamp time of 73 ± 20 min and average pump time of 97 ± 24 min. Off-pump surgery was performed using the Medtronic Octopus Stabilizer (Medtronic, Dublin, Ireland) and FlowThru coronary shunts (Synovis, Birmingham, UK) of adequate size. 3 proximal anastomoses of SVG and 2 distal anastomoses of arterial grafts were revised due to abnormal TTFM readings (flow< 20 ml/min and/or PI > 5). During the revision of the venous grafts, the external stent was slightly compressed to enable graft exposure and was then re-expanded following revision. Intra and post-operative data are  summarized in Table 3.

**Table 2:** Grafts distribution according to the coronary target

	N (%)
**LIMA graft**	**118**
LIMA to LAD	99 (83.8)
LIMA to CRX	0 (0)
LIMA to OM	7 (5.9)
LIMA to Diagonal	8 (6.7)
LIMA to IR	4 (3.3)
**RIMA graft**	**23**
RIMA to LAD	3 (13)
RIMA to CRX	0 (0)
RIMA to OM	11 (47.8)
RIMA to Diagonal	1 (4.3)
RIMA to IR	8 (34.7)
**Externally stented SVG**	**110**
SVG to CRX	11 (10)
SVG to OM	28 (25.4)
SVG to diagonal	16 (14.5)
SVG to IR	11 (10)
SVG to PDA	31 (28.1)
SVG to RCA	13 (11.8)
**None stented SVG**	**16**
SVG to CRX	1 (6.2)
SVG to OM	6 (37.5)
SVG to diagonal	7 (43.7)
SVG to IR	0 (0)
SVG to PDA	2 (12.5)
SVG to RCA	0 (0)

**Table 3:** Intra and post-operative data

	N (%)
Concomitant valve or aortic surgery	13 (13%)
Off-pump CABG	51 (50%)
IMA-LAD grafts	102 (100%)
Use of RIMA	23 (23%)
Single vein graft (pts.)	57 (56%)
Sequential vein grafts (pts.)	45 (44%)
Mean surgery time (min.)	187 ± 37
Mean cross clamp time (min.)	73 ± 20
Mean pump time (min.)	97 ± 24
Mean extubation time (h.)	8.2 ± 6
Mean Intensive Care Unit Stay (days)	1.6 ± 1.12
Mean in Hospital Stay (days)	7.4 ± 1.5

*CABG* coronary artery bypass grafting, *LAD* left anterior descending artery, *LIMA* left internal mammary artery, *RIMA* right internal mammary artery

One patient experienced a perioperative myocardial infarction (0.9%) due to occlusion of the Left Internal Mammary Artery (LIMA)-LAD graft and was treated by PCI. Of note, this patient had undergone an endarterectomy of the LAD during operation. One patient experienced a transient ischemic attack on postoperative day 4 (0.9%). No other in-hospital events were recorded.

All patients completed CT-angiography assessment at 6–12 months’ time window. As shown in Table 4, all the arterial grafts were patent. In addition, 98% of the externally stented SVG (2 occlusions) and 87.5% of the non-stented grafts (2 occlusions) were patent. No differences in SVG patency were observed between the off- and on- pump CABG groups or the different grafting techniques (single versus sequential). 90% of the externally stented SVG had uniform lumen compared to 37% of the non-stented SVG. No CT-angiograohy artifacts were observed in the externally stented SVG and the lumen was well defined in all grafts. CT-angiography imaging demonstrated well the spatial positioning of the SVG and the stents, especially in multi-anastomoses sequential setting, and no kinking or unexpected twisting were observed.

**Table 4:** Grafts patency and uniformity assessed by CT angoigrpahy

	N (%)	N (%)	N (%)
	Patent (< 50% stenosis)	Patent (> 50% stenosis)	Occluded
LIMA-LAD (99)	99 (99%)	1 (1%)	
RIMA (24)	23 (100%)		
Exteranlly stented SVG:			
- Single SVG (65)	63 (97%)		2 (3%)
- Sequential SVG (45)	45 (100%)		
None stented SVG:			
- Single SVG (17)	14 (87.5%)		2 (12.5%)
- Sequential SVG (0)			
SVG Uniformity at CT angiography:			
Exteranlly stented SVG (110)			
- Uniform lumen	99 (90%)		
- Non-uniform lumen	11 (10%)		
None Stented SVG (17)			
- Uniform lumen	6 (37%)		
- Non-uniform lumen	10 (63%)		

Clinical follow-up was completed for all patients with a mean duration of 20 months (range 6–54 months). During the follow-up period, no additional MACCE events were recorded.

## Discussion

This report describes the use of external stents for SVG during routine CABG practice which includes off pump CABG, sequential grafting and the use of multiple external stents per patient. To the best of our knowledge, this is the first time that external stents performance is being evaluated as part of routine practice. Our main finding is that external stenting is safe and can be seamlessly integrated into the routine CABG practice, with minimal changes to the standard grafting technique. No traction on the anastomoses was observed during stent positioning and the ability to shape the device together with the graft in-situ allowed for precise positioning of the vein curvatures in multiple anastomoses sequential settings.

The etiology of early SVG failure is multifactorial and related to surgical skill, graft quality, harvesting technique and the size of the coronary vascular bed [4, 5]. As shown in Table 5, contemporary trials reported early (up to 1 year after CABG) SVG patency rates between 80 to 85% in on pump CABG and less than 80% in off pump CABG [14–19]. The accuracy of CT angiography in detecting the presence and severity of coronary and graft disease was shown to be comparable to invasive coronary angiography in several trials. Studies related to 64-slices CTangiography technology reported sensitivity values ​​of 93–99% and specificity of 95–97% with a negative predictive value of 99% [20]. One potential explanation for the high early patency rates observed in our study is the use of an open rather than endoscopic harvesting technique. Recent meta analysis has shown that endoscopic vein harvesting, even in experienced hands, may reduce short and intermediate term SVG patency due to trauma to the vein and endothelial damage [21]. In addition, all procedures were performed by senior surgeons who used TTFM to identify technical errors prior to closing the chest. As previously reported and confirmed also in our study, TTFM allows detection of technical problems at the level of the anastomoses, leading to the revision of 2–4% of bypass grafts with a consequent reduction in early graft failure and its related clinical events [22]. Our low MACCE rates may be attributed in part to the high early patency rates of the venous and the arterial grafts. As shown in the PREVENT IV trial, 18 months after CABG, death, myocardial infarction and revascularization rate were significantly higher in patients who experienced at least one early SVG failure compared to those who didn’t (26% compared to 1.8% respectively) [16].

**Table 5:** SVG patency rates at 1 year

	# of patients	# of vein grafts	SVG Patency rate at 1 year (%)
Desai et al. (2004) [﻿^14^﻿]	440	440	86.4
Goldman et al. (2004) [15]	799		84
PREVENT IV (2005) [16]	951	2242	73.5
Cho et al. (2006) [17]	109	227	82.4
Kim et al. (2008) [18]	349	121	76
Shroyer et al. (2009) [19]	685	1262 Off-pump	76.6
	686	1339 On-pump	83.8

The majority of externally stented SVGs demonstrated uniform lumen. This finding is in correlation with previous preclinical and clinical reports on the beneficial effect of external stents on lumen uniformity flow patterns and the protective effect of laminar flow against the development of SVG intimal hyperplasia [10–13]. This is in contrast to areas with low and oscillatory wall shear stress which are more prone to endothelial dysfunction, thrombus formation and more aggressive vascular disease [23].

Our study is part of intensive clinical research aiming to overcome the Achilles heel of CABG. SVG preservation solutions, no-touch harvesting technique and external stents are all attempts to change the natural history of SVG. Even if it does not immediately translate to hard clinical endpoints, mitigating well validated surrogate SVG disease markers such as intimal hyperplasia and lumen irregularities is another important step in the journey to improve the clinical outcome of surgical revascularization.

This study has a single arm with relatively low sample size and limited follow up duration. The lack of proper randomization between the stented and non-stented SVG and the fact that two experienced coronary surgeons performed all the procedures, limit the generalizability of the results. Larger, well designed, randomized trials with 5–10 years of follow up are required to further define the role of external stents in CABG and its clinical benefit.

## Conclusions

The use of external stents in a complex, ‘real world’ CABG setting is technically feasible and safe with acceptable early patency rates and good clinical outcomes. According to our experience, the device can be integrated into routine CABG practice with a minimal learning curve.

## Data Availability

The datasets used and/or analysed during the current study are available from the corresponding author on reasonable request.

## Abbreviations

SVG: Saphenous Vein Graft
CABG: Coronary Artery Bypass Grafting
MACCE: Major Adverse Cardiac and Cerebrovascular Events
IMA: Internal Mammary Artery
LAD: Left Anterior Descending Artery
LIMA: Left Internal Mammary Artery
RIMA: Right Internal Mammary Artery
BIMA: Bilateral Internal Mammary Artery
TTFM: Transit Time Flow Measurement
